# Maintenance of methylation profile in imprinting control regions in human induced pluripotent stem cells

**DOI:** 10.1186/s13148-022-01410-8

**Published:** 2022-12-28

**Authors:** A. Pham, C. Selenou, E. Giabicani, V. Fontaine, S. Marteau, F. Brioude, L. David, D. Mitanchez, M. L. Sobrier, I. Netchine

**Affiliations:** 1 INSERM, Centre de recherche Saint Antoine, Sorbonne Université, 75012 Paris, France; 2 Hôpital Armand Trousseau, Service de néonatologie, APHP, 75012 Paris, France; 3 Hôpital Armand Trousseau, Endocrinologie moléculaire et pathologies d’empreinte, APHP, 75012 Paris, France; 4 Institut de Cardiométabolisme et Nutrition, Sorbonne Université, 75013 Paris, France; 5 CHU Nantes, Inserm, CR2TI, Université de Nantes, 44000 Nantes, France; 6 CHU Nantes, Inserm, CNRS, BioCore, Université de Nantes, 44000 Nantes, France; 7 Hôpital Bretonneau, Service de néonatologie, CHRU de Tours, 37000 Tours, France

**Keywords:** Induced pluripotent stem cells, Imprinting disorders, Parental imprinting, Methylation, Silver–Russell syndrome, Temple syndrome, Chondrogenic differentiation

## Abstract

**Background:**

Parental imprinting is an epigenetic mechanism that leads to monoallelic expression of a subset of genes depending on their parental origin. Imprinting disorders (IDs), caused by disturbances of imprinted genes, are a set of rare congenital diseases that mainly affect growth, metabolism and development. To date, there is no accurate model to study the physiopathology of IDs or test therapeutic strategies. Human induced pluripotent stem cells (iPSCs) are a promising cellular approach to model human diseases and complex genetic disorders. However, aberrant hypermethylation of imprinting control regions (ICRs) may appear during the reprogramming process and subsequent culture of iPSCs. Therefore, we tested various conditions of reprogramming and culture of iPSCs and performed an extensive analysis of methylation marks at the ICRs to develop a cellular model that can be used to study IDs.

**Results:**

We assessed the methylation levels at seven imprinted loci in iPSCs before differentiation, at various passages of cell culture, and during chondrogenic differentiation. Abnormal methylation levels were found, with hypermethylation at 11p15 *H19/IGF2*:IG-DMR and 14q32 *MEG3/DLK1*:IG-DMR, independently of the reprogramming method and cells of origin. Hypermethylation at these two loci led to the loss of parental imprinting (LOI), with biallelic expression of the imprinted genes *IGF2* and *DLK1*, respectively. The epiPS™ culture medium combined with culturing of the cells under hypoxic conditions prevented hypermethylation at *H19/IGF2*:IG-DMR (ICR1) and *MEG3/DLK1*:IG-DMR, as well as at other imprinted loci, while preserving the proliferation and pluripotency qualities of these iPSCs.

**Conclusions:**

An extensive and quantitative analysis of methylation levels of ICRs in iPSCs showed hypermethylation of certain ICRs in human iPSCs, especially paternally methylated ICRs, and subsequent LOI of certain imprinted genes. The epiPS™ culture medium and culturing of the cells under hypoxic conditions prevented hypermethylation of ICRs in iPSCs. We demonstrated that the reprogramming and culture in epiPS™ medium allow the generation of control iPSCs lines with a balanced methylation and ID patient iPSCs lines with unbalanced methylation. Human iPSCs are therefore a promising cellular model to study the physiopathology of IDs and test therapies in tissues of interest.

**Supplementary Information:**

The online version contains supplementary material available at 10.1186/s13148-022-01410-8.

## Background

Parental imprinting is an epigenetic mechanism that leads to the monoallelic expression of a subset of genes depending on their parental origin [1]. Differential methylation of imprinting control regions (ICRs) is among the most studied mechanisms controlling such monoallelic expression. Imprinting disorders (IDs), caused by disturbances of imprinted genes, are a set of rare congenital diseases that mainly affect growth and metabolism [2]. Silver-Russell syndrome (SRS, MIM#180860), characterized by intra-uterine and postnatal growth retardation [3], is one such rare disease. Loss of methylation at the paternal *H19/IGF2*:IG-DMR (differentially methylated region) (also called ICR1) at chromosome 11p15 is the principal molecular anomaly identified in these patients. Other molecular etiologies may be at the origin of SRS such as total or partial deletions of *H19/IGF2*:IG-DMR or loss of function mutations of *IGF2*. All these molecular defects lead to a decrease in *IGF2* expression responsible for the SRS phenotype. Other imprinting regions are involved in human IDs, such as 15q11-13 for Prader–Willi syndrome (PWS, MIM#176266) and 14q32 for Temple syndrome (TS, MIM#616222), leading to diseases with overlapping features, regardless of the genomic region affected [4].

The low expression of imprinted genes in human tissues available for biological research, such as leucocytes and fibroblasts, is a major limit for studying IDs and testing therapies at the cellular level [4]. In addition, tissues of interest in these diseases, such as cartilage, bone, liver, and brain, are not accessible. For all these reasons, many groups have attempted to develop cellular models to approach the mechanisms underlying the physiopathology of IDs [5–8].

Human induced pluripotent stem cells (iPSCs) are a promising cellular approach to model human diseases and complex genetic disorders due to their ability to self-renew and to differentiate into all three germ layers in culture [9, 10]. Human iPSCs can be directly reprogrammed from somatic cells [11] and have been derived from patients with certain IDs [5, 7, 10, 12, 13]. However, epigenetic modifications and the erasure of imprinting can appear during the reprogramming process and subsequent culture of iPSCs [6, 8, 9]. Therefore, we extensively analyzed methylation marks at imprinted loci in iPSCs before and after adjustment of the culture medium to develop a cellular model of IDs with balanced methylation.

## Results

All the methylation studies have been assessed by using the allele-specific methylated multiplex real-time quantitative PCR technique using designed and validated TaqMan MGB probes (covering two to three CpG islands each) and primers as previously described [14]. The normal band of methylation indices represented by a shaded area corresponds to normal values obtained in control individuals’ leukocytes and validated for IDs diagnosis in our molecular biology laboratory [14–16].

### Methylation of ICRs in urine-derived iPSCs and during chondrogenic differentiation

**Fig. 1:**
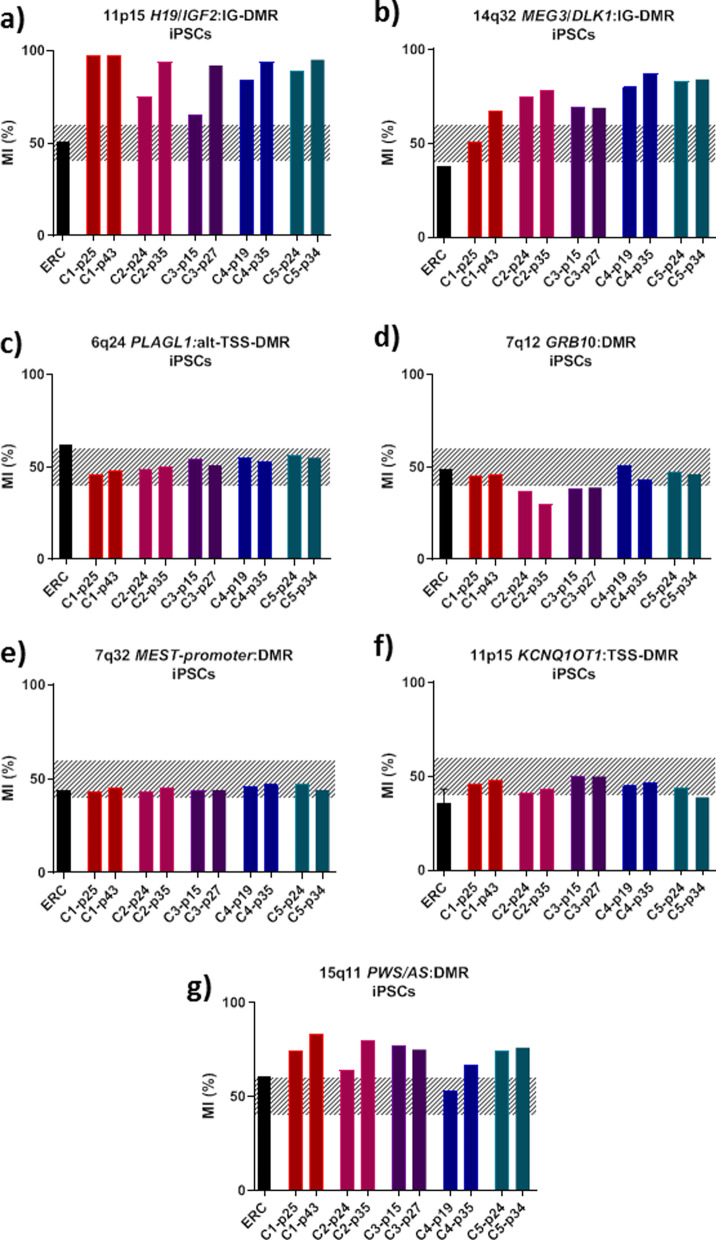
Methylation levels of five iPSC clones (C1–5) at 11p15 *H19/IGF2*:IG-DMR (**a**), *14q32 DLK1/MEG3:IG-DMR* (**b**), 6q24 *PLAGL1*:alt-TSS-DMR (**c**), 7q12 *GRB10*:DMR (**d**), 7q32 *MEST* promoter DMR (**e**), 11p15 *KCNQ1OT1*:TSS-DMR (**f**) and 15q11 *PWS/AS*:DMR (**g**)*.* The hatched part represents the average normal MI area for each locus. *MI* methylation index, *p* passage, *ERC* epithelial renal cells

**Fig. 2:**
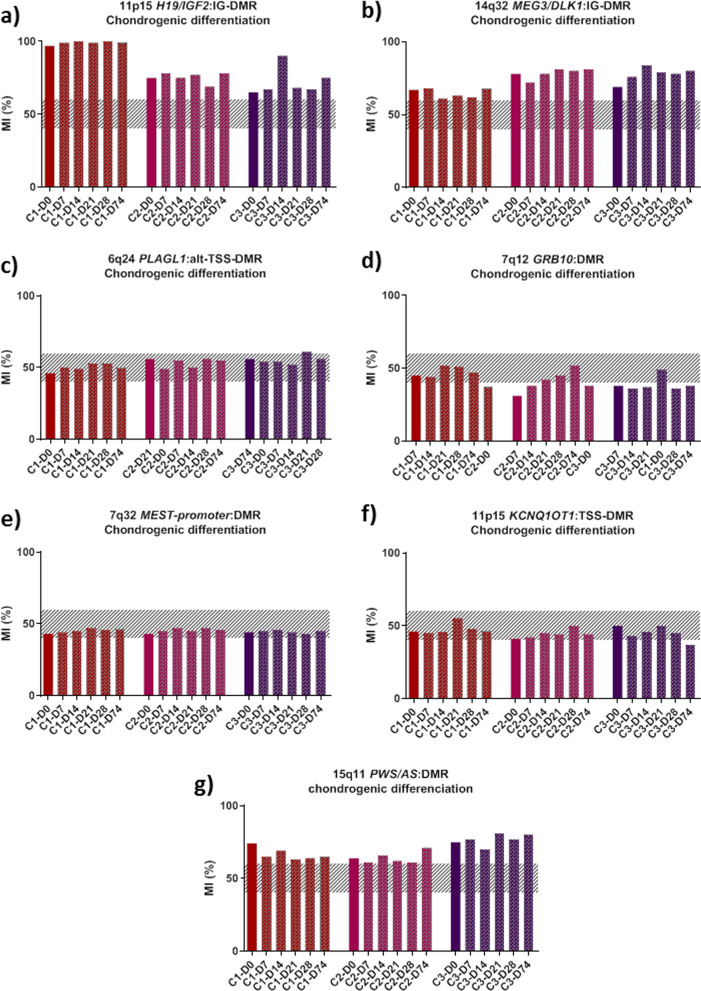
Methylation levels of three iPSC clones (C1–3) at 11p15 *H19/IGF2*:IG-DMR (**a**), *14q32 DLK1/MEG3:IG-DMR* (**b**), 6q24 *PLAGL1*:alt-TSS-DMR (**c**), 7q12 *GRB10*:DMR (**d**), 7q32 *MEST* promoter DMR (**e**), 11p15 *KCNQ1OT1*:TSS-DMR (**f**) and 15q11 *PWS/AS*:DMR (**g**) before (D0), during (D7-D28), and after (D74) chondrogenic differentiation. The hatched part represents the average normal MI area for each locus. *D* day, *MI* methylation index

At first, we studied methylation at seven imprinted loci in epithelial renal cells (ERCs) before iPSC reprogramming and in five available controls urine-derived iPSC clones (C1-5) at various passages of cell culture. Some clones showed hypermethylation at *H19/IGF2*:IG-DMR (ICR1), 14q32 *MEG3/DLK1*:IG-DMR and 15q11 *PWS/AS*:DMR (Fig. 1a, b, g). Methylation was balanced for all the urine-derived iPSCs at 6q24 *PLAGL1*:alt-TSS-DMR, 7q12 *GRB10*:DMR, the 7q32 *MEST* promoter DMR, and 11p15 *KCNQ1OT1*:TSS-DMR (ICR2) (Fig. 1c–f). Then, we investigated influence of iPSCs differentiation on methylation. For that purpose, three of the clones were cultured in chondrogenic differentiation medium after different numbers of passages (25 for C1, 24 for C2 and 15 for C3). The efficiency of chondrogenic differentiation is depicted in Additional file 1: Figure SD1, showing an increase in the quantitative expression of the specific markers *Aggrecan*, *COL2A1*, *COL10A1,* and *SOX9* at day 28 of chondrogenic differentiation (Figure SD1, in Additional file 1). The methylation levels remained unchanged during and after chondrogenic differentiation. None of the tested clones showed balanced methylation at all loci (Fig. 2).

### Extensive analysis of the *IGF2/H19* imprinting control region

**Fig. 3:**
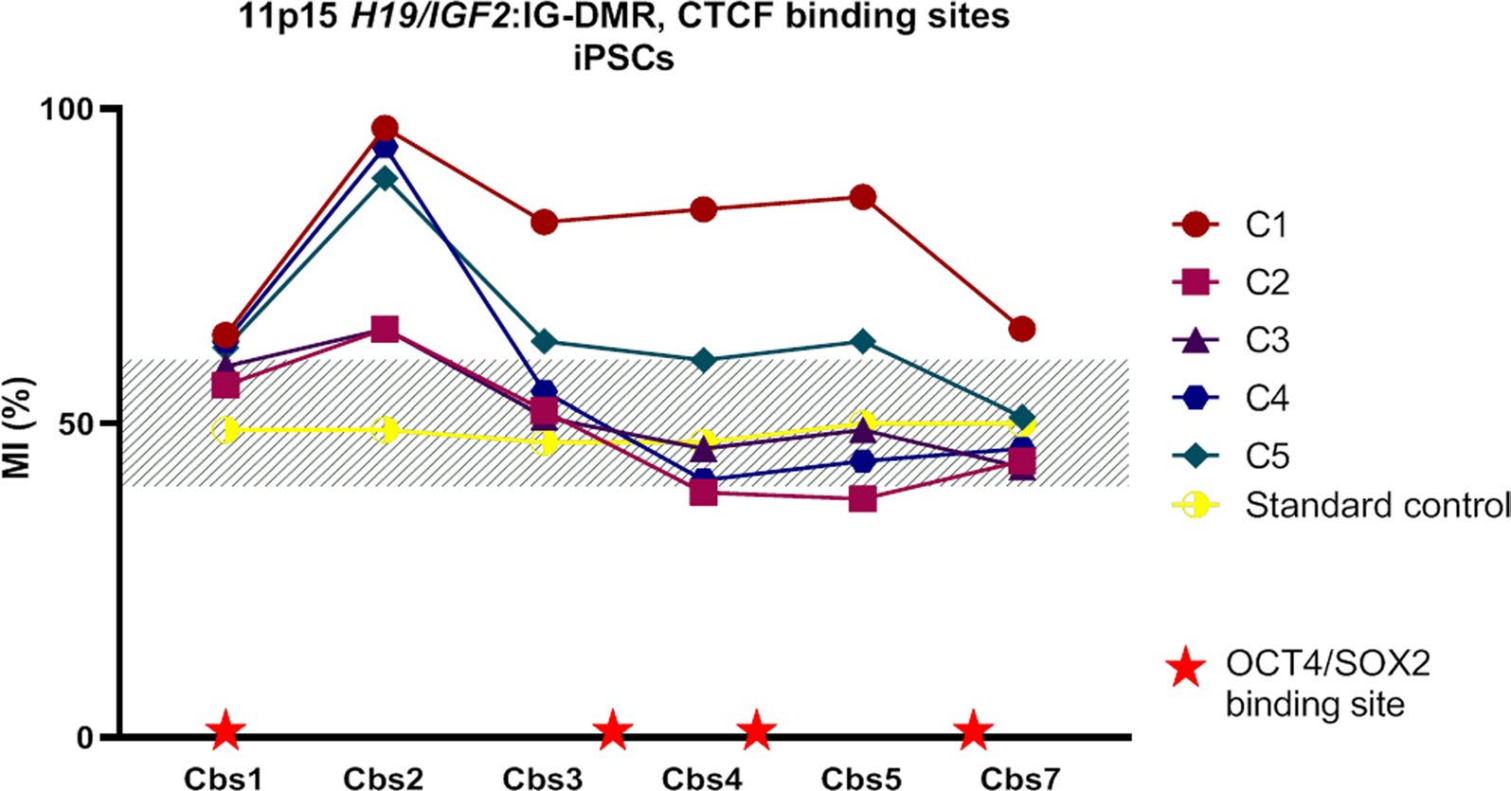
Extensive investigation of methylation levels at the 11p15 *H19/IGF2*:IG-DMR in urine derived iPSCs. The methylation level was studied at six CTCF binding sites. *MI* methylation index, *Cbs* CTCF binding site

We studied the methylation profile of the *H19/IGF2*:IG-DMR (ICR1) domain at six CTCF binding sites (CBS 1, 2, 3, 4, 5, 7) in the urine-derived iPSCs clones before chondrogenic differentiation (Fig. 3). One clone (C1) was hypermethylated at all CBSs and the others were almost all normal at all CBSs, except at CBS2, for which all clones showed an abnormal and higher level of methylation.

### Imprint relaxation

**Fig. 4:**
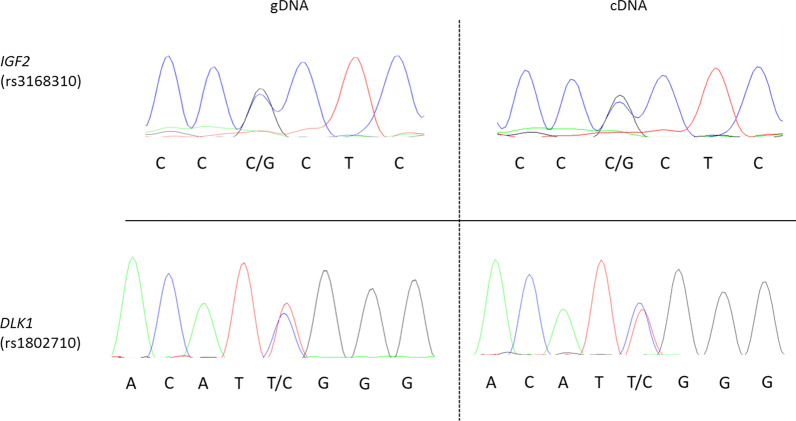
Electrophoregrams of genomic DNA (gDNA) and complementary DNA (cDNA) for one clone (Clone 1) carrying polymorphisms in *IGF2 (rs3168310)* and *DLK1 (rs1802710)*, allowing the analysis of allelic-type expression of these genes

We identified polymorphisms in *IGF2* (rs3168310) and *DLK1* (rs1802710) in the genomic DNA of C1, allowing us to study the mono or biallelic expression of these imprinted genes. Both *IGF2* and *DLK1* showed biallelic expression (Fig. 4), indicating a loss of imprinting (LOI) at these two loci, results in accordance with the hypermethylation of the corresponding ICRs.

### Impact of the method and cell type on iPSC reprogramming

We studied the methylation levels at *H19/IGF2*:IG-DMR (ICR1) in 10 iPSC clones from different somatic cells of origin reprogrammed by various methods and cultured in mTeSR1 or KSR feeder-free medium. All iPSC clones showed aberrant hypermethylation at 11p15 ICR1, independently of the reprogramming method (mRNA transfection or episomal vector) or somatic cell of origin (skin fibroblast or peripheral blood mononuclear cells-PBMCs) (Table 1).

**Table 1:** Methylation levels at 11p15 *H19/IGF2*:IG-DMR of 10 iPSC clones (C6–C15). C6–C12 were reprogrammed from skin fibroblasts and C13–C15 from peripheral blood mononuclear cells (PBMCs). The reprogramming of somatic cells into iPSCs was carried out using mRNA transfection (C6–C11) or an episomal vector (C12–C15). iPSCs have been cultivated in classical culture conditions

Clone	Somatic cells of origin	Technic of reprogramming	Culture conditions	11p15 *H19/IGF2*:IG-DMR, MI (%)
C6	Skin fibroblast	mRNA transfection	KSR feeder-free medium Normoxia	96
C7	Skin fibroblast	mRNA transfection		80
C8	Skin fibroblast	mRNA transfection		70
C9	Skin fibroblast	mRNA transfection		68
C10	Skin fibroblast	mRNA transfection		89
C11	Skin fibroblast	mRNA transfection		66
C12	Skin fibroblast	Episomal vector	mTeSR1 feeder-free medium Normoxia	67
C13	PBMC	Episomal vector		64
C14	PBMC	Episomal vector		67
C15	PBMC	Episomal vector		68

### Impact of culture conditions on methylation at imprinted loci

As similar hypermethylation was observed independently of the cell type of origin and reprogramming method, we suspected the culture conditions to have a major influence. We tested this hypothesis using the culture medium we adapted epiPS™” (mTeSR1 supplemented with ascorbic acid) and culturing the iPSCs under conditions of hypoxia or normoxia. We carried out the reprogramming experiments from PBMCs, which are easier to obtain, cultivate, and amplify than ERCs.

**Fig. 5:**
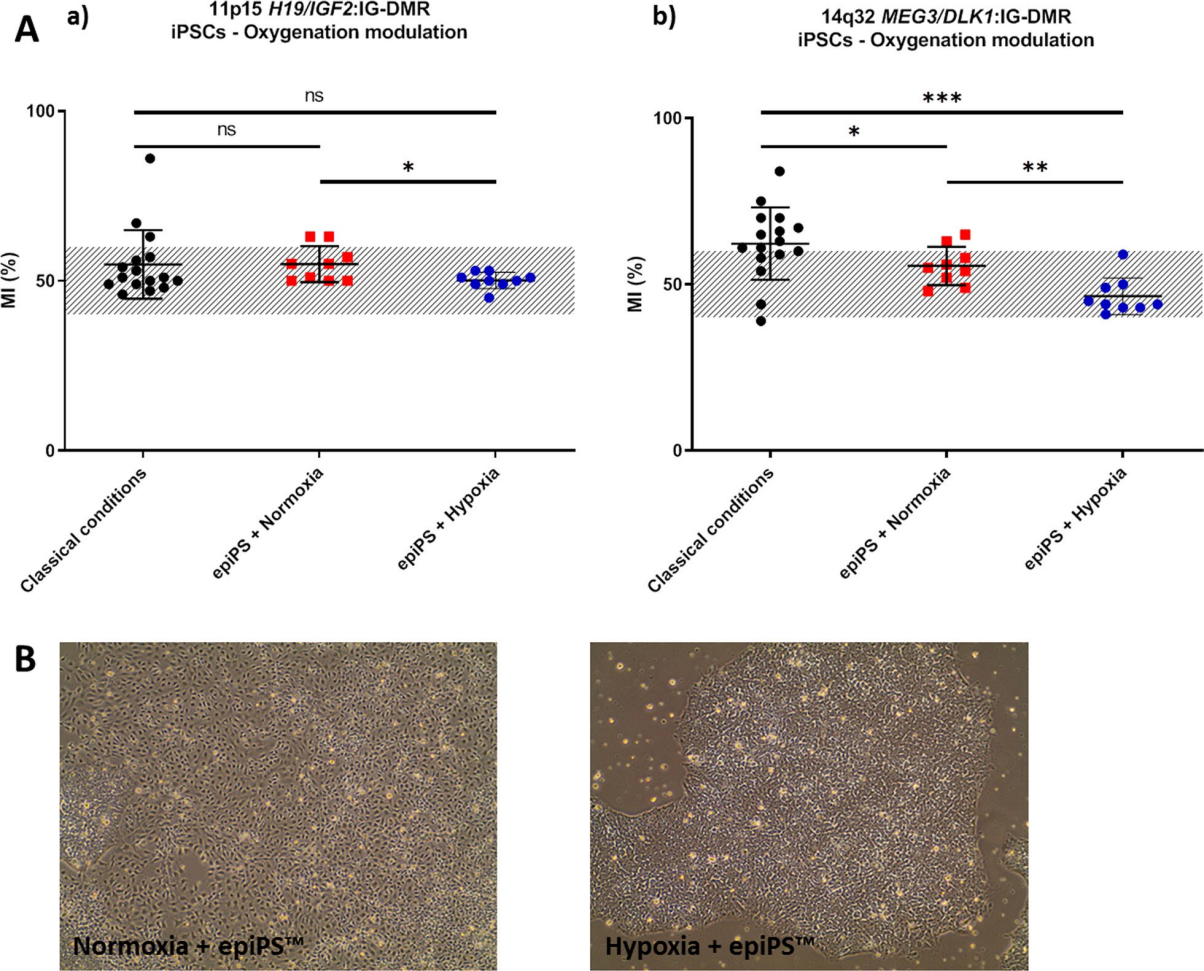
**A** Methylation levels of 16 ERC-derived iPSCs clones at passage 4 cultivated in mTeSR medium in normoxia (classical conditions) and nine PBMC-derived iPSC clones at passage 5 (nine clones of iPSCs from one control) cultured with epiPS™ medium in normoxia or hypoxia at 11p15 *H19/IGF2*:IG-DMR (ICR1) (a) and *14q32 DLK1/MEG3:IG-DMR* (b). The hatched part represents the average normal MI area for each locus. Analysis using Wilcoxon test (comparison of epiPS™ in Normoxia and epiPS™ in hypoxia) and Mann–Whitney test (comparison classical conditions with epiPS™ in Normoxia and epiPS™ in Hypoxia). **B** Morphology of iPSC cultured with epiPS™ medium in hypoxia or normoxia conditions. The iPSC clone cultivated with epiPS™ medium in normoxia displays a critical spontaneous differentiation unlike the same iPSC clone cultivated with epiPS™ medium in hypoxia. Same clone at passage 5, enlargement × 10

First, we tested the effect of ascorbic acid on methylation levels at 11p15 *H19/IGF2*:IG-DMR and 14q32 *MEG3/DLK1*:IG-DMR loci in 16 iPSCs clones cultivated in normoxia in mTeSR medium (which are the classical culture conditions for iPSCs) and on nine clones cultivated in epiPS™ medium in normoxia. In classical culture conditions, most of iPSCs clones displayed an hypermethylation in 14q32 *MEG3/DLK1*:IG-DMR unlike iPSCs cultivated in epiPS™ medium in normoxia where methylation is balanced and stable (Fig. 5A). However, iPSCs cultivated in epiPS™ in normoxia exhibited a frequent and complete spontaneous differentiation (Fig. 5B). Secondly, we cultured the same nine clones in epiPS™ medium under hypoxia and observed both balanced methylation at 11p15 *H19/IGF2*:IG-DMR and 14q32 *MEG3/DLK1*:IG-DMR loci and optimal pluripotency status without frequent spontaneous differentiation (Fig. 5A, B).

**Fig. 6:**
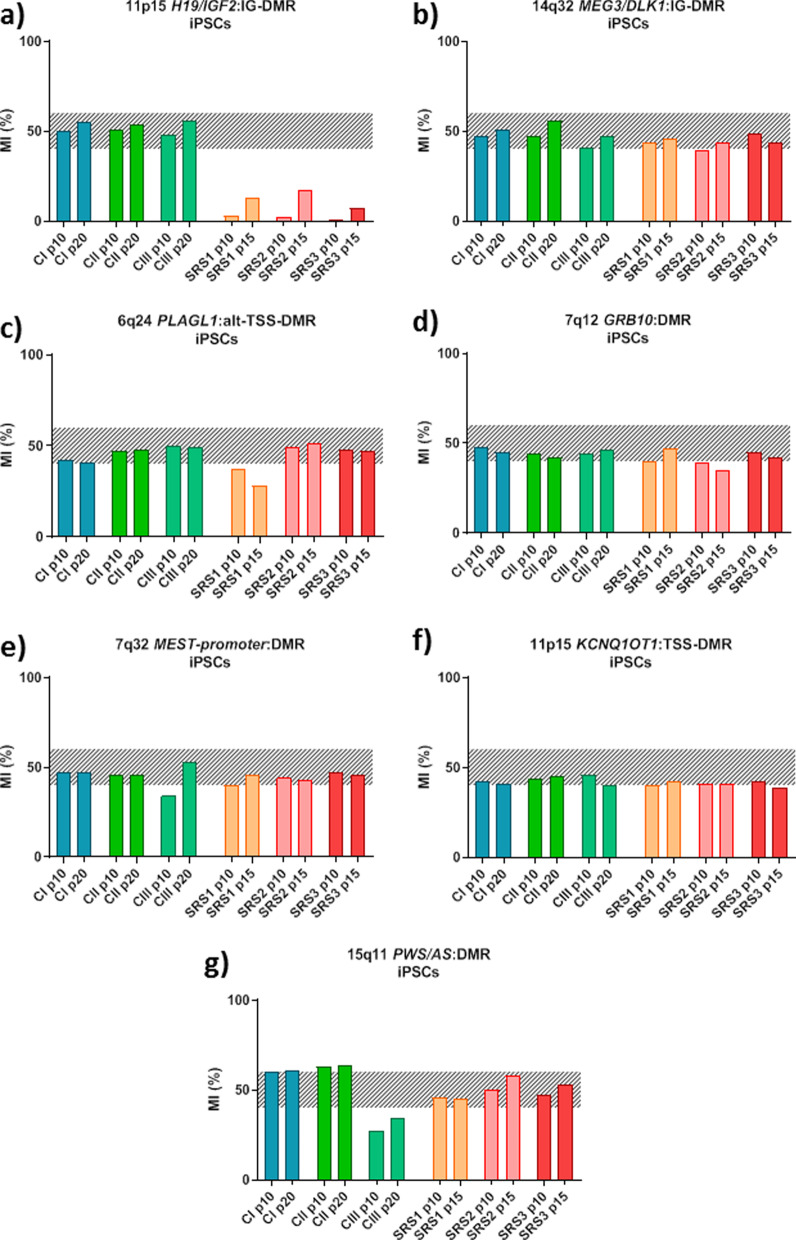
Methylation levels of six PBMC-derived iPSC clones (Three clones of iPSCs from one control individual and from one patient with SRS) cultured with the epiPS™ medium and under hypoxia at 11p15 *H19/IGF2*:IG-DMR (ICR1) (**a**), *14q32 DLK1/MEG3:IG-DMR* (**b**), 6q24 *PLAGL1*:alt-TSS-DMR (**c**), 7q12 *GRB10*:DMR (**d**), 7q32 *MEST* promoter DMR (**e**), 11p15 *KCNQ1OT1*:TSS-DMR (**f**) and 15q11 *PWS/AS*:DMR. The hatched part represents the average normal MI area for each locus. *MI* methylation index, *p* passage

**Fig. 7:**
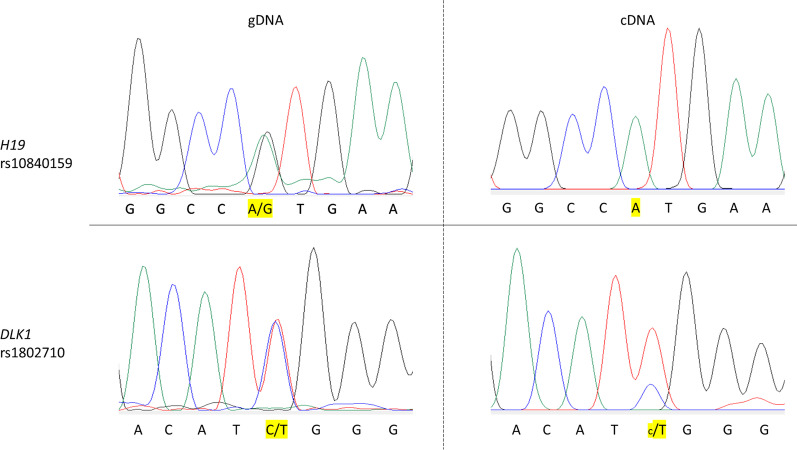
Electrophoregrams of genomic DNA (gDNA) and complementary DNA (cDNA) for clone III cultivated in epiPS™ medium in hypoxia. The clone carrying polymorphisms in *H19* (rs10840159) and *DLK1* (rs1802710), allowing the analysis of allelic-type expression of these genes

These results clearly demonstrate the synergistic effect of hypoxia and epiPS™ medium on the maintenance of pluripotency and balanced methylation, respectively. In a second time, three clones were cultured in hypoxia until 20 passages and IM was measured at 11p15 *H19/IGF2*:IG-DMR and 14q32 *MEG3/DLK1*:IG-DMR (Fig. 6a, b), as well as in five other imprinted loci (Fig. 6c–g). For the major part of loci studied, the methylation stayed between 45–60% and remained stable from the passage 10 to 20. The imprinted loci 15q11 *PWS/AS*:DMR seems to be at the limit of the hypermethylation for the clone I at p10 and displays a slight hypermethylation in the clone I at p20, II at p10 and II at p20. Surprisingly, the clone III at p10, displays loss of methylation at 7q32 *MEST* promoter DMR, and 15q11 *PWS/AS*:DMR loci; this hypomethylation is corrected at passage 20 except for the locus 15q11 *PWS/AS*:DMR in clone III. After identifying SNP in the imprinted genes *H19* (rs10840159) and *DLK1* (rs1802710) in clone III, we studied their allelic expression. *DLK1* has a strong bias toward one allele corresponding to parental imprinting maintenance (Fig. 7).

Under these new culture conditions, another reprogramming was carried out on PBMCs from a SRS patient with a heterozygous deletion of the paternal *H19/IGF2*:IG-DMR region. This is an isolated genetic anomaly, so the other loci are not affected. As expected, this three patient's iPSCs clones show loss of methylation at the 11p15 *H19/IGF2*:IG-DMR and balanced methylation at the 14q32 *MEG3/DLK1*:IG-DMR at passage 10 and 15 (Fig. 6a, b). All other loci show balanced methylation for all three clones except for the 6q24 *PLAGL1*:alt-TSS-DMR locus where one clones exhibits a loss of methylation. (Fig. 6c–g). The allelic expression could not be performed due to the absence of SNP in *IGF2*, *H19*, *DLK1* imprinted genes in this patient’s DNA.

In conclusion, the results show that the best culture conditions are the epiPS™ culture medium used in synergy with hypoxia, allowing to obtain iPSCs with a globally balanced methylation at the imprinted loci and an optimal pluripotency status.

## Discussion

We performed an extensive quantitative analysis of methylation levels of several ICRs to assess whether parental imprinting is maintained during the reprogramming and culture of human iPSCs. We found abnormal methylation levels at the two imprinted loci governed by a paternally methylated DMR in human feeder-free iPSCs derived from controls with no IDs, independently of the reprogramming method or somatic cell of origin. Hypermethylation at 11p15 *H19/IGF2*:IG-DMR and 14q32 *MEG3/DLK1*:IG-DMR led to the loss of parental imprinting, with biallelic expression of the imprinted genes *IGF2* and *DLK1*, respectively. To overcome this, we modified the culture strategy by developing epiPS™ medium that we combined with hypoxia culture to correct aberrant methylation at these two ICRs. Analysis of the methylation of seven ICRs showed that these culture conditions prevented the hypermethylation at most ICRs associated to IDs in iPSCs generated from both control and SRS patient PBMC while retaining the qualities of proliferation and pluripotency and, thus, offering a promising perspective to develop a human cellular model of ID.

Our results and those of several other previous studies suggest a trend toward hypermethylation of certain ICRs in human iPSCs when cultured in the classic conditions, especially paternally methylated ICRs, and LOI of certain imprinted genes. Several groups have reported aberrant DNA methylation or LOI at 11p15 *H19/IGF2*:IG-DMR and 14q32 *MEG3/DLK1*:IG-DMR in mouse and human pluripotent stem cells, which persisted after differentiation into various cell types, but they focused exclusively on these two loci [9, 17–19]. We tested these and several other loci and found that imprinted genes governed by a paternally methylated DMRs appear to be more frequently affected by LOI in iPSCs than those controlled by a maternally methylated DMR [9]. Bar and Benvenisty hypothesized that paternally imprinted methylated regions are more sensitive to aberrations during reprogramming due to different mechanisms for protecting imprinted regions from demethylation and de novo methylation in paternally versus maternally methylated regions [9]. We thus studied the methylation profile at the *H19/IGF2*:IG-DMR (ICR1) domain at six CTCF binding sites (CBS) and found the highest level of methylation in the most distal CBS from OCT4 and SOX2 binding sites, CBS2, which are known to protect the maternal allele against de novo methylation during early embryogenesis consistent with the hypothesis of Bar and Benvenisty [20, 21]. On the other hand, several groups have suggested that clones with aberrant hypermethylation are selected during the culture of iPSCs due to the upregulation of growth-promoting genes, such as *IGF2*, or the silencing of growth-restricting genes, which promote self-renewal, growth, and survival of iPSCs in culture [22, 23].

In contrast to embryonic stem cells (ESCs), global hypermethylation of gDNA of the entire genome has been described in iPSCs at early passages (i.e., p3–p5), which then disappeared during subsequent passages [24–26]. By contrast, we found that abnormal methylation at ICRs persisted during in vitro culture up to 43 passages and the differentiation of iPSCs. Recent studies in mouse and human iPSCs have suggested that DNA methylation of the genome in these cells is highly dynamic, cycling between de novo methylation and its erasure [27, 28]. However, such turnover was not observed in imprinted regions, which is consistent with our results [28].

Several iPSC models derived from patients with IDs have been described in the literature. For example, Chamberlain and Burnett derived iPSCs from patients with Prader–Willi syndrome due to a paternally inherited deletion of chromosome 15q11-q13 and Chang et al*.* derived iPSCs from patients with Beckwith–Wiedemann syndrome with paternal uniparental disomy of chromosome 11. In these studies, human iPSCs derived from controls and patients showed the same methylation patterns as the fibroblast lines from which they were derived [5–7]. However, these authors studied methylation levels at only one locus for Chamberlain (15q11 *PWS/AS*:DMR) and two loci for Chang (*H19/IGF2*:IG-DMR and 11p15 *KCNQ1OT1*:TSS-DMR) which may explain why they did not observe the methylation abnormalities specific to reprogramming and culture of iPSCs under classical conditions. Indeed, Okuno et al*.* demonstrated that a fully methylated status for chromosome 15q11 in fibroblasts could be reversed to a partially unmethylated status in at least some of the iPSCs after reprogramming and Nazor et al*.* demonstrated a loss of methylation at the 15q11 locus in a subset of control iPSC lines [29, 30]. As imprinted genes are co-regulated and organized in an imprinted-gene network, abnormal methylation at one locus can modify the expression of imprinted genes of other imprinted loci, even if the methylation at the corresponding ICR is not affected but also the expression of non-imprinted genes [4, 31–35].

By using the epiPS™ medium (a medium supplemented with ascorbic acid) during the culture of human iPSCs and under hypoxia, we prevented aberrant methylation of ICRs and retained proliferation and pluripotency qualities of iPSCs. Stadtfeld et al*.* have shown that ascorbic acid treatment can efficiently protect the 14q32 imprinted region from LOI during the derivation of mouse iPSCs [36]. They suggested that ascorbic acid may prevent the loss of H3K4 methylation at the maternal IG-DMR during reprogramming and then prevent the recruitment of Dnmt3a, which is essential for 14q32 *MEG3/DLK1*:IG-DMR DNA methylation. However, they did not examine the methylation levels at other ICRs. More recently, Arez et al. have shown that the addition of ascorbic acid during the reprogramming of murine cells stabilizes the methylation of *H19/Igf2* and *Dlk1*-*Dio3* regions which were hypermethylated without this addition [37]. Moreover, although it has long been accepted that hypoxia enhances the generation of iPSCs, its combination with ascorbic acid has never been reported [38]. Our extensive and quantitative analysis of ICR methylation levels in iPSCs derived from controls and cultured with the addition of ascorbic acid and under hypoxia allowed to obtain a significant number of iPSCs clones with balanced methylation at imprinted loci tested, implying, moreover, a synergistic effect of these two elements. Indeed, in normoxia with the addition of ascorbic acid, not only some clones acquire a light but significative hypermethylation during the passages but also, iPSCs lose their ability to proliferate and tend to differentiate spontaneously. We suspected that the high concentration of ascorbic acid plays a major role in this phenomenon in keeping with the fact that, ascorbic acid is a well-known differentiating factor. Various studies have demonstrated the beneficial effect of hypoxia on the pluripotency status of stem cells [38].

Here, we report, for the first time, an extensive analysis of methylation levels of several ICRs involved in human IDs in iPSCs. Because of the organization of imprinted loci in an imprinted-gene network, the level of methylation needs to be studied at least in all imprinted loci implicated in human imprinted disorders before using iPSCs as a cellular model of IDs. We show that the culture of iPSCs under hypoxia prevents aberrant hypermethylation of ICRs in iPSCs derived from controls and patient with ID.

## Conclusions

Through an extensive and quantitative analysis of the methylation levels of ICRs in iPSCs, we found hypermethylation of certain ICRs in human iPSCs, particularly paternally methylated ICRs, and subsequent LOI of certain imprinted genes. The addition of ascorbic acid included in epiPS™ medium during the culture of iPSCs under hypoxia prevented the hypermethylation of ICRs; epiPS™ culture medium allowing to maintain balanced methylation at imprinted loci and hypoxia conditions allowing to maintain pluripotency of iPSCs. As balanced methylation is maintained during the reprogramming and culture of iPSCs in these conditions, human iPSCs are a promising cellular model to study the physiopathology of IDs and test therapies, after differentiation, in tissues of interest. As imprinted genes are organized in an imprinted-gene network, methylation levels need to be studied at all ICRs involved in imprinting diseases in human before using iPSCs as a cellular model of disease.

## Methods

### Human urine-derived iPSC reprogramming

Urine-derived iPSCs from controls were kindly provided by the iPSC core facility of Nantes Université supported by Biogenouest and IBiSA.

*Isolation of ERCs (epithelial renal cells) and reprogramming.* Urine samples were collected and ERCs isolated by the iPSC core facility (INSERM, CNRS, UNIV Nantes, CHU Nantes, France) from urine samples and cultures, as previously described [39]. Briefly, cells were seeded on Matrigel-coated wells on day − 1 in their usual culture media and transfected daily, from day 0 to day 10, with 625 ng of an mRNA cocktail (38% Oct4, 11.4% Sox2, 12.7% Klf4, 10.1% Lin28, 12.7% Myc, 10.1% Nanog, 5.1% nGFP, Milenyi Biotec) in Pluriton media (Reprocell, Glasgow, G20 0XA UK) supplemented with 4 ng/ml FGF2 (Peprotech, Neuilly sur Seine, France) and 200 ng/ml B18R (eBioscience, ThermoFisher scientific). From day 11, cells were cultured in Pluriton media supplemented with 4 ng/ml FGF2. Colonies were picked and expanded on feeders in KSR + FGF2 media or directly on Matrigel-coated dishes in TeSR1 or iPS Brew. The expression of pluripotency genes (*Oct4, Sox2, Nanog*) was verified by qPCR (Additional file 2: Figure SD2 and Additional file 3: Table SD3). Assays were performed to assess the ability of each iPSC clone to differentiate into the three germ line lineages [39]. The karyotype of each iPSC cell line was normal.

### iPSC culture

Human urine-derived iPSCs were initially cultured in feeder-free KSR medium (DMEM/F-12, 20% KnockoutTM serum replacement, 1% non-essential amino acids, 1% Glutamax, 50 µM 2-mercaptoethanol, and 10 ng/ml fibroblast growth factor 2 [Peprotech Neuilly sur Seine, France]) at the iPSC core facility (INSERM, CNRS, UNIV Nantes, CHU Nantes, France). They were mechanically passaged by cutting colonies with a needle. All cells were cultured at 37 °C under 20% O_2_ and 5%CO_2_. Then, iPSCs were cultured at the Institute of Cardiometabolism and Nutrition (ICAN, F-75013 Paris, France). Colonies were picked and expanded in mTeSR1 (Stemcell technologies, Vancouver, BC, Canada) on Matrigel matrix-coated plates. The iPSCs were passed manually once a week using a Lynx microscope (Vision Engineering, New Milford, CT, USA), and the culture medium was changed daily. The cells were cultured at 37 °C under 5% CO_2_ and 5% O_2_.

### Chondrogenic differentiation

Chondrogenic differentiation of iPSCs was performed using a published protocol [40]. Briefly, iPSCs were subjected to differentiation by changing the medium to a mesendodermal differentiation medium (DMEM/F12 with 10 ng/ml Wnt3a [R&D Systems], 10 ng/ml Activin A [R&D], 1% ITS [Invitrogen], 1% FBS [Invitrogen]) (day 0). At day 3, the medium was changed to basal medium (DMEM with 1% ITS and 1% FBS) supplemented with 50 μg/ml ascorbic acid (Nacalai), 10 ng/ml BMP-2 (Osteapharma), 10 ng/ml GDF5, 10 ng/ml TGFβ (Peprotech), and 10 ng/ml FGF-2. Fourteen days after starting the differentiation of the iPSCs (day 14), the cartilaginous nodules were physically separated from the bottom of the dishes to form particles, which were then transferred to a suspension culture in 3.5-cm petri dishes. To increase proliferation, 50 μg/ml ascorbic acid (Nacalai), 10 ng/ml BMP-2 (Osteapharma), 10 ng/ml GDF5, and 10 ng/ml TGFβ (Peprotech) was added to the chondrogenic medium from day 3 to day 14. The medium was changed to conventional medium (DMEM with 10% FBS) on day 42. The medium was changed every 2 to 7 days.

### Human fibroblast-derived iPSCs and PBMC-derived iPSC reprogramming

Fibroblast- or PMBC-derived iPSCs were kindly provided by the Institute of Cardiometabolism and Nutrition (ICAN, F-75013 Paris, France) or the iPSC core facility (INSERM, CNRS, UNIV Nantes, CHU Nantes, France) after reprogramming by various methods (episomal vector, mRNA transfection) as previously described [39, 41, 42].

### Peripheral blood mononuclear cell (PBMC) culture and reprogramming with ascorbic acid

After the collection of PBMCs from control (obtained through the “Établissement Français du Sang” (EFS) according to the current ethical rules) and SRS patient (Comité de Protection des Personnes 18/56), they were allowed to proliferate for 7 days in blood medium (StemPro34 SFM medium with 100 µg/mL SCF, 100 µg/mL FLT3, 100 µg/mL IL3, 100 µg/mLIL6, and 54 U/µL EPO). For reprogramming, 2 × 10^5^ cells were cultured in 24-well plates with a virus cocktail using the Sendaï 2.0 CytoTuneiPS reprogramming kit (Life Technologies) in either 5% CO_2_, 20% O_2_, and 75% N_2_ (normoxia condition) or 5% CO_2_, 5% O_2_, and 90% N_2_ (hypoxia condition). The virus was removed 24 h later (centrifugation at 300 × *g* for 7 min) and the cells transferred to 12-well plates in 1 ml blood media. After 3 days in culture, the cells were transferred to matrix gel-coated 6-well plates. At 7 days post-transduction, the cells were cultured in epiPS™ medium (mTeSR1 medium completed with ascorbic acid (50 µg/ml)) and half-was renewed daily. Fifteen days post-transduction, the iPSC clones appeared, were picked, and cultured in epiPS™ medium.

### Immunofluorescence staining

iPSCs were cultured in four-well culture slides (Corning) for 3 days and then fixed in paraformaldehyde (4%) and permeabilized in blocking/permeabilization buffer (2% BSA, 0.5% Triton-X-100 in PBS) for 45 min and then incubated overnight at 4 °C with primary antibodies diluted in blocking/permeabilization buffer. The cells were washed three times in PBS and incubated with Alexa-conjugated secondary antibodies and DAPI, both diluted 1:1000 in blocking/permeabilization buffer, for 45 min at room temperature. The images were acquired using an epifluorescence microscope (Eclipse TE300, Nikon, Amsterdam, the Netherlands). The following antibodies were used: rabbit anti-Nanog (#4903S, 1:200, Cell Signaling-Ozyme, Beverly, MA, USA), rabbit anti-Oct4 (#3576-100, 1:200, Biovision, Cliniscience, Mountain View, CA, USA), rabbit anti-Sox2 (#AB5603, 1:200, Millipore, Ballerica, MA, USA), mouse anti-Tra-1-60 (#MAB4360, 1:100, Millipore), mouse anti-Tra-1-81 (#MAB4381, 1:100, Millipore), and mouse anti-SSEA4 (#sc-21704, 1:100, Santa Cruz, Dallas, TX, USA) (Figure SD2, in Additional file 2).

### Karyotype analysis

Conventional cytogenetic studies on cell cultures at passage 20 were performed by Trousseau Hospital to verify chromosomal integrity. Fifteen metaphases were analyzed (Figure SD2, in Additional file 2).

### Embryoid body formation and scorecard

Generated iPSCs were grown on Matrigel, dispensed into small clumps with a cell scraper, and cultured in suspension for 10 days in TeSR-E6 medium (Stemcell). At day 8, RNA was isolated from the embryoid bodies (Ambion). A scorecard kit (Life Technologies) was used to evaluate the gene expression of the three germ layers. The scorecard plate was analyzed using a StepOneplus™ system real-time PCR (Life Technologies) (Figure SD2, in Additional file 2).

### Alkaline phosphatase

Alkaline phosphatase activity was measured using a detection kit (Sigma-Aldrich) and performed following the manufacturer's instructions (Figure SD2, in Additional file 2).

### RNA extraction and reverse transcription

RNA was extracted from iPSCs before and on days 7, 14, 21, 28, and 74 of chondrogenic differentiation. Total RNA was extracted using the NucleoSpin miRNA Kit for the isolation of small and large RNA (Macherey–Nagel, France) with DNase treatment. Both DNA and RNA were quantified using a DS-11 spectrophotometer (DeNovix). cDNA was synthesized from long RNA using the miScript PCR System (Qiagen, France) and used for quantitative PCR.

### Quantitative expression of chondrogenic differentiation-specific markers

Quantitative expression of chondrogenic differentiation-specific markers (*Aggrecan*, *COL2A1*, *COL10A1*, *SOX9*) was assessed for all samples using SyBR Select Master Mix (Applied Biosystems, ThermoFisher Scientific) and a Light Cycler LC480 instrument (Roche LifeSciences). The primers are listed in Table SD4 in Additional file 4.

### DNA extraction

DNA was extracted from iPSCs before and on days 7, 14, 21, 28, and 74 of chondrogenic differentiation using an in-house protocol after cell lysis by a salting-out procedure, as previously described [43, 44].

### Bisulfite treatment of DNA

Sodium bisulfite treatment of DNA converts all unmethylated cytosine residues to uracil residues. The methylated cytosine residues are unaffected. This process generates C/T polymorphisms, which can be used to distinguish between the methylated and unmethylated allele. Genomic DNA (400 ng) was treated with sodium bisulfite using the EZ DNA Methylation Lighting kit (Zymo Research, USA), according to the manufacturer’s instructions. Genomic DNA was eluted using 40 μl RNase-free H_2_O and conserved at − 20 °C.

### TaqMan allele-specific methylated multiplex real-time quantitative PCR (ASMM RTQ-PCR) and methylation analysis.

The methylation status of seven imprinted loci (seven DMRs: 6q24 *PLAGL1*:alt-TSS-DMR, 7q12 *GRB10*:DMR, 7q32 *MEST* promoter DMR, 11p15 *H19/IGF2*:IG-DMR, 11p15 *KCNQ1OT1*:TSS-DMR, 14q32 *MEG3/DLK1*:IG-DMR, 15q11 *PWS/AS*:DMR) was assessed by ASMM RTQ-PCR, as previously described [14]. The methylation index (MI) at each locus was assigned by calculating the ratio between the methylated and unmethylated alleles as follows: (amount of methylated allele/sum of both methylated and unmethylated alleles) × 100. The ASMM RTQ-PCR primers and probes sequences are provided in Supplementary Methods (Table SD5, in Additional file 5).

### gDNA and cDNA sequencing

To assess the mono or biallelic expression of imprinted genes, two single nucleotide polymorphisms from the *H19* (rs217727) and *DLK1* (rs1802710) gDNA and cDNA were sequenced by standard Sanger sequencing by Eurofins Genomics (Germany). The primers used for gDNA and cDNA amplification and sequencing are provided in Table SD6, in Additional file 6). The sequencing products were then analyzed using Sequencing Analysis 5.2. Generated and available iPSCs lines and sex of donors are resumed in Table SD7, in Additional file 7. All iPSCs lines have been derived from somatic cells of male individuals.

### Statistics

Data in the figures are presented as means. All graphs were generated and statistical analysis performed using GraphPad Prism 6 (USA).

## Supplementary Information

**Additional file 1: Figure SD1.** Quantitative expression of chondrogenic markers in chondrogenic iPSCs by qPCR at day 28 of differentiation (C1-D28, C2-D28, C3-D28). C: clone, D: day. iPSCs is D0 of each clone.

**Additional file 2: Figure SD2.** Pluripotency of iPSC cell lines derived from control (CI, CII) and patient (SRS1, SRS2) and associated karyotypes. A. Immunostaining of iPSCs with antibodies directed against the pluripotency markers NANOG, OCT4, SOX2 (red), TRA1-60, TRA1-81 and SSEA-4 (green). Nuclei were stained with DAPI (blue). B. Normal karyotyping. C. Positive alkaline phosphatase staining. D. RT-PCR showing the expression of the pluripotency genes of clones CI, CII, SRS1 and SRS2 relative to a control iPSC cell line. E. Scorecards. TM analysis assessing pluripotency and trilineage differentiation.

**Additional file 3: Table SD3.** Primer sequences for pluripotent genes. F: forward, R: reverse.

**Additional file 4: Table SD4.** Primer sequences for qPCR of chondrogenic differentiation-specific markers. F: forward, R: reverse.

**Additional file 5: Table SD5.** Primer and probe sequences for ASMM RTQ-PCR. F: forward, R: reverse, M: methylated allele, UM: unmethylated allele.

**Additional file 6: Table SD6.** Primers used for gDNA and cDNA amplification and sequencing. F: forward, R: reverse.

**Additional file 7: Table SD7.** Generated and available iPSCs lines and sex of donors. All iPSCs lines have been derived from somatic cells of male individuals.

## Data Availability

The datasets used and/or analyzed during the current study are available from the corresponding author on reasonable request.

## Declarations

### Invention declaration

The iPSCs culture with epiPS™ medium under hypoxia constitutes a provisional patent deposit at the European Patent Office by Professor Netchine's team and the IHU-ICAN on July 13, 2022, under the file number EP22306056.
