# Supplementary figures and images for: Maintenance of methylation profile in imprinting control regions in human induced pluripotent stem cells
Source: Clin Epigenetics. 2022 Dec 28;14:190. doi: 10.1186/s13148-022-01410-8 (PMC9798676; doi:10.1186/s13148-022-01410-8)

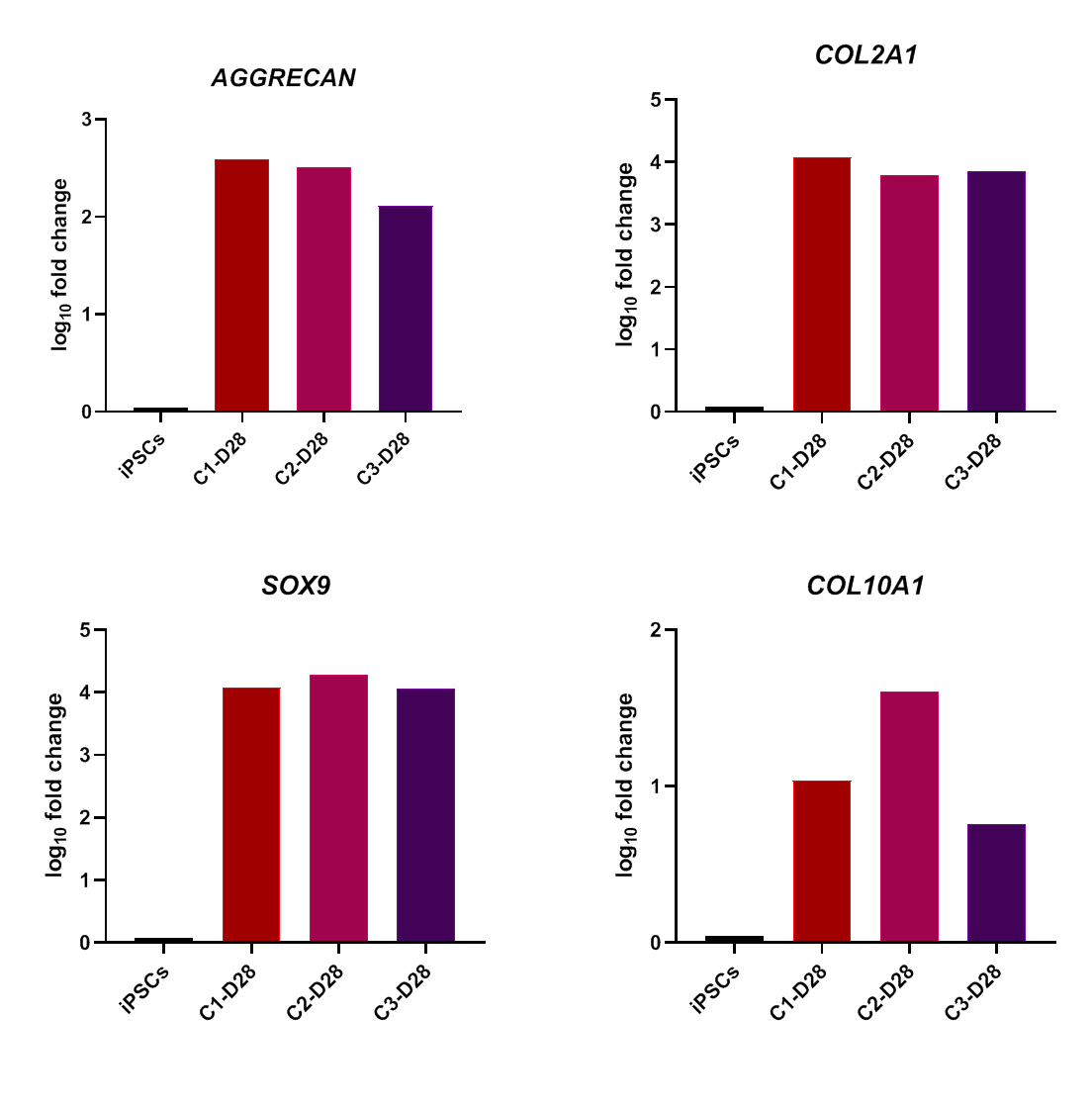

Supplement: Supplementary file 1 — Additional file 1: Figure SD1. Quantitative expression of chondrogenic markers in chondrogenic iPSCs by qPCR at day 28 of differentiation (C1-D28, C2-D28, C3-D28). C: clone, D: day. iPSCs is D0 of each clone. [file 13148_2022_1410_MOESM1_ESM.png]

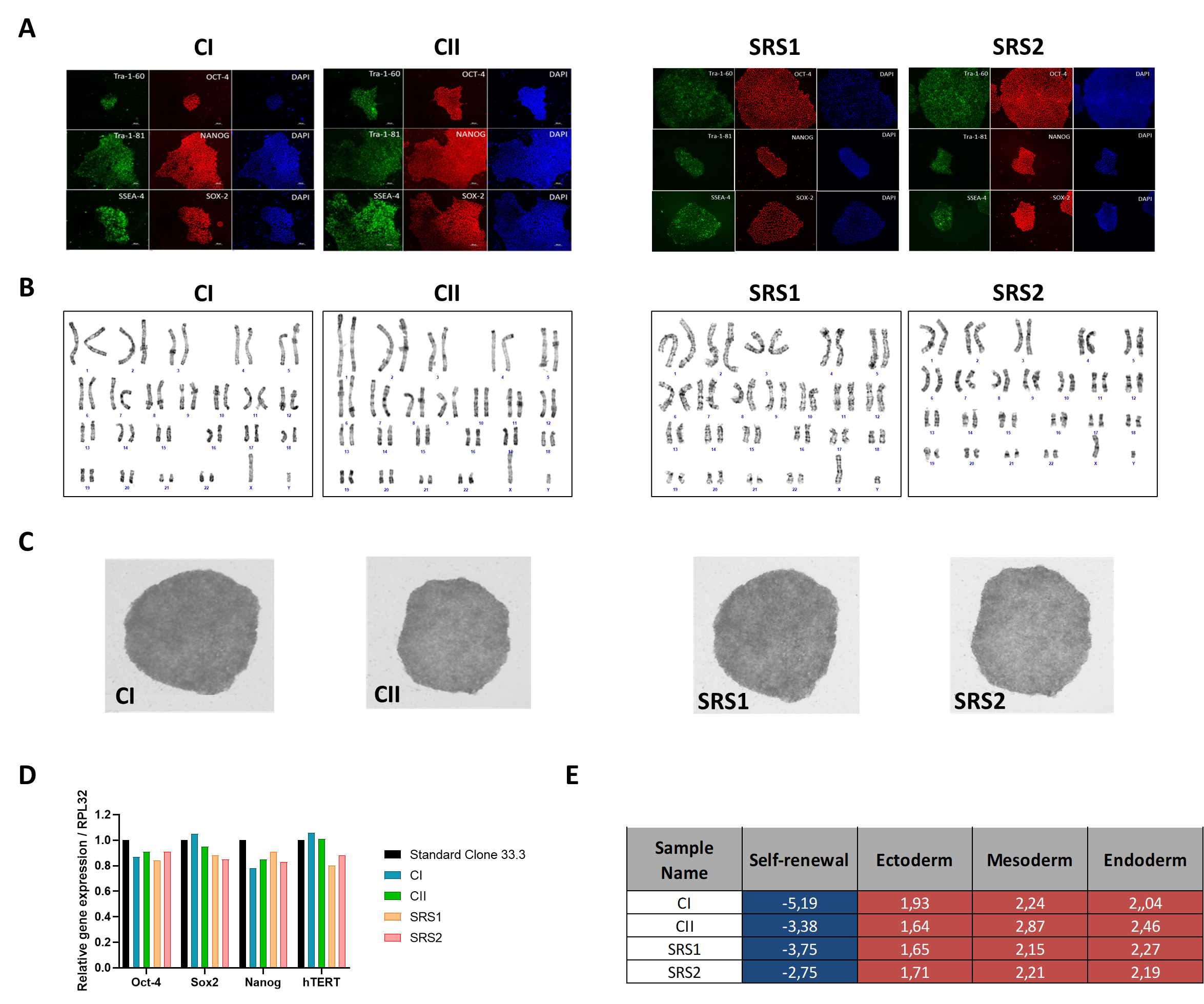

Supplement: Supplementary file 2 — Additional file 2: Figure SD2. Pluripotency of iPSC cell lines derived from control (CI, CII) and patient (SRS1, SRS2) and associated karyotypes. A. Immunostaining of iPSCs with antibodies directed against the pluripotency markers NANOG, OCT4, SOX2 (red), TRA1-60, TRA1-81 and SSEA-4 (green). Nuclei were stained with DAPI (blue). B. Normal karyotyping. C. Positive alkaline phosphatase staining. D. RT-PCR showing the expression of the pluripotency genes of clones CI, CII, SRS1 and SRS2 relative to a control iPSC cell line. E. Scorecards. TM analysis assessing pluripotency and trilineage differentiation. [file 13148_2022_1410_MOESM2_ESM.png]
